# A novel tool for evaluating non-cognitive traits of doctor of physical therapy learners in the United States

**DOI:** 10.3352/jeehp.2018.15.19

**Published:** 2018-08-17

**Authors:** Marcus Roll, Lara Canham, Paul Salamh, Kyle Covington, Corey Simon, Chad Cook

**Affiliations:** 1 Department of Orthopedics, Duke University, Durham, NC, USA; 2 Department of Physical Medicine and Rehabilitation, School of Medicine, University of Colorado Anschutz Medical Campus, Aurora, CO, USA; 3 Krannert School of Physical Therapy, University of Indianapolis, Indianapolis, IN, USA; Hallym University, Korea

**Keywords:** School admission criteria, Physical therapists, Statistical factor analysis, United States

## Abstract

**Purpose:**

The primary aim of this study was to develop a survey addressing an individual’s non-cognitive traits, such as emotional intelligence, interpersonal skills, social intelligence, psychological flexibility, and grit. Such a tool would provide beneficial information for the continued development of admissions standards and would help better capture the full breadth of experience and capabilities of applicants applying to doctor of physical therapy (DPT) programs.

**Methods:**

This was a cross-sectional survey study involving learners in DPT programs at 3 academic institutions in the United States. A survey was developed based on established non-proprietary, non-cognitive measures affiliated with success and resilience. The survey was assessed for face validity, and exploratory factor analysis (EFA) was used to identify subgroups of factors based on responses to the items.

**Results:**

A total of 298 participants (90.3%) completed all elements of the survey. EFA yielded 39 items for dimensional assessment with regression coefficients < 0.4. Within the 39 items, 3 latent constructs were identified: adaptability (16 items), intuitiveness (12 items), and engagement (11 items).

**Conclusion:**

This preliminary non-cognitive assessment survey will be able to play a valuable role in DPT admissions decisions following further examination and refinement.

## Introduction

Although there are many metrics for determining an applicant’s cognitive traits, few metrics are known to assess a prospective student’s non-cognitive abilities [1]. Non-cognitive traits encompass a prospective student’s behaviors, motivations, and personality [2]. Emotional intelligence, which encompasses managing one’s own emotions and interpersonal relationships, has been correlated with improved grade point average in nursing programs [3]. In medical students, academic success has also been correlated with emotional intelligence [4]. Improved patient outcomes and adherence to treatment plans have been shown to occur when healthcare practitioners possess desirable non-cognitive traits, such as integrity, motivation, strong interpersonal and intrapersonal communication skills, and the ability to collaborate with colleagues [5]. With the absence of a non-cognitive metric, admissions committees struggle to identify students who will thrive in their educational setting and ultimately develop into excellent clinicians [6].

The purpose of this study was to develop a preliminary tool to identify the non-cognitive traits of prospective learners. Using preexisting validated items from non-cognitive questionnaires, we implemented qualitative and quantitative processes to identify items that were succinct and discriminative of non-cognitive traits. The overarching goal of this study was to initiate the development of a non-cognitive metric that physical therapy admissions committees can use to evaluate an individual’s full breadth of potential. Subsequently, non-cognitive traits can be assessed in future studies to identify associations with academic and clinical excellence and leadership.

## Methods

### Ethical statement

The study was approved by the institutional ethics boards of Duke University (#83516), the University of Colorado (#17-1228), and the University of Indianapolis (#0828) after receiving informed consent from the subjects.

### Study design

This was a cross-sectional survey study involving doctor of physical therapy (DPT) programs at 3 academic institutions.

### Instrument development

Two researchers (CS and CC) performed a literature search to identify key non-proprietary, non-cognitive measures associated with success and resilience. The intent was to include a wide range of standardized non-cognitive measurement sources that reflected emotional intelligence, interpersonal skills, social intelligence, resilience, psychological flexibility, and/or grit. Questionnaires that were well represented in the literature and targeted specific constructs were sought out. The following 6 questionnaires were identified: (1) the Schutte Self Report Emotional Intelligence Test [7], (2) the Interpersonal Reactivity Index (IRI) [8], (3) the Intolerance of Uncertainty Scale (IUS) [9], (4) Measuring Social Intelligence (MSI) [10], (5) the Psychological Flexibility Questionnaire [11], and (6) the Short Grit Scale (Grit-S) [12].

The Schutte Self Report Emotional Intelligence Test is a 33-item measure of emotional intelligence based on the work of Salovey and Mayer’s emotional intelligence model [13] and then refined by Schutte et al. [7] in 1998. The test requires respondents to determine how much each statement describes them, utilizing a 5-point Likert scale ranging from 5 (strongly agree) to 1 (strongly disagree). Higher response scores correspond to higher emotional intelligence, and individuals with higher emotional intelligence have shown better abilities to manage stress in both academic and clinical settings [14]. The test demonstrated a high internal consistency among college students (Cronbach alpha= 0.87) [7]. Validation studies have demonstrated correlations with the theoretical constructs of alexithymia, attention to feelings, clarity of feelings, mood repair, optimism, and impulse control. The scale has also been demonstrated to predict the grades of first-year collegiate students, despite not assessing cognitive ability [7].

The IRI is a 28-item measure of empathy. Empathy is a foundational trait for healthcare practitioners to develop meaningful relationships with their patients, and it should be assessed and cultivated in healthcare professionals [15,16]. The IRI uses a 5-point scale ranging from 4 (describes me very well) to 0 (does not describe me well). There are 4 subscales within the IRI that address both cognitive and non-cognitive components of empathy. The 4 scales include: (1) perspective taking, (2) empathic concern, (3) fantasy, and (4) personal distress. The IRI has demonstrated moderate levels of internal consistency for females (Cronbach alpha= 0.70–0.78) and males (Cronbach alpha= 0.75–0.78) [16].

The IUS is a scale consisting of 27 items that revolve around the idea that “uncertainty is unacceptable, reflects badly on a person, and leads to frustration, stress, and the inability to take action” [9]. The IUS items are scored using a 5-point Likert scale describing statements as ranging from 5 (entirely characteristic of me) to 1 (not at all characteristic of me). Both the initial IUS version in French by Freeston and colleagues in 1994 and an English translation by Buhr and Dugas [9] in 2002 demonstrated excellent internal consistency (Cronbach alpha= 0.94) and good test-retest reliability (r= 0.74). Tolerance of uncertainty is essential in healthcare professions and has been linked to leadership potential in physicians [17], how clinicians make decisions with their patients, and how providers exchange information to build therapeutic relationships [18]. Conversely, higher intolerance of uncertainty in physicians has been associated with increased charges for services [19] and medical students with a higher intolerance of uncertainty avoid working with underserved populations [20].

The MSI scale contains 21 items, and respondents utilize a 5-point scale ranging from 4 (very often) to 0 (never) in descriptions of social scenarios. Three constructs are included in the MSI scale: (1) manipulation, (2) empathy, and (3) social irritability. It is thought that social intelligence and emotional intelligence are very closely related [21]. Social intelligence includes how one perceives and expresses emotion in new situations, both interpersonally and intrapersonally [10]. Social intelligence is also linked with the development of goals and motivation [10]. Interdisciplinary teamwork is essential for competent patient care [22]. Effective teams contain individuals who are respectful, active listeners, and seek the knowledge of teammates [23]. These necessary behaviors and attitudes that drive successful teams can be improved during education and are a significant part of healthcare educational curricula [22,23].

The Psychological Flexibility Questionnaire is a 20-item scale that measures 5 factors: (1) positive perception of change, (2) characterization of self as flexible, (3) self-characterization as open and innovative, (4) a perception of reality as dynamic and changing, and (5) a perception of reality as multifaceted [11]. The questionnaire uses a 5-point Likert scale ranging from 6 (very much) to 1 (not at all) to determine the degree to which a respondent is characterized by each statement. The questionnaire has demonstrated high reliability (Cronbach alpha= 0.918). Individuals with high psychological flexibility have demonstrated a greater ability to work towards goals in stressful environments and a lower risk of burnout [24].

The Grit-S is an 8-item scale designed to measure “perseverance and passion toward long-term goals” [25]. The Grit-S was based on original work by Duckworth et al. [25] in 2007 and then refined to its current form in 2009 by Duckworth and Quinn [12]. Items are scored on a 5-point scale ranging from 5 (very much like me) to 1 (not at all like me). Grit and resilience have received prominent attention in health education research [26]. Grit has been associated with success in both academic and professional environments [25]. The scale has also demonstrated high internal consistency and predictive validity [12,25].

The 6 identified questionnaires were obtained and each item was transposed to a document and de-identified. The face validity of the survey was assessed by 2 researchers (CS and CC) through blinded-rater agreement, and individual scale items were excluded based on: (1) the ease of determining the desired response of the question, (2) grammar and ease of understanding the items, and (3) the potential ceiling and floor effects of the question. In short, the researchers were concerned that the directionality of many of the items would allow individuals to “game” the questions and answer in a way that they thought they should, rather than based on how they felt. The agreement for the initial removal process was 75.0%. After consensus, 75 items were removed from the original 143 items, leaving a total of 68 items for dimensional analysis.

### Participants

The survey was sent to students in the first- and second-year DPT programs at 3 institutions: Duke University Doctor of Physical Therapy Program, University of Colorado Doctor of Physical Therapy Program, and University of Indianapolis Krannert School of Physical Therapy. Duke University is a private university in Durham, North Carolina with a Carnegie classification of a doctoral university: highest research activity. The University of Colorado is a public university in Aurora, Colorado with a Carnegie classification of a doctoral university: highest research activity. The University of Indianapolis is a private university in Indianapolis, Indiana with a Carnegie classification of master’s colleges and universities: larger program.

The partnership of the institutions in this study revolved primarily around an interest in improving admissions processes. Ideally, researchers wanted to capture survey results from a wide range of students in diverse geographic locations and at both public and private institutions. Descriptive statistics for the cohorts were shared, including age at time of application, year in the DPT program, and sex (Table 1). These were the only descriptive statistics shared among institutions. Each institution maintained additional identifiable information for their respective cohorts.

### Administration of the instrument

All surveys were administered through Qualtrics software (Qualtrics, Provo, UT, USA). Each institution used the Qualtrics email function to maintain identified data for the survey respondents.

The survey was first presented to the Duke University cohorts. Researchers oriented the first- and second-year students to the purpose of the survey, and it was stressed that the survey would have no repercussions on academic standing or other adverse effects. The survey was formulated and sent to the first- and second-year classes at Duke University on March 21, 2017. A follow-up reminder email was sent on March 27, 2017 to students who had not completed the survey. The survey closed on April 1, 2017, with 144 of a possible 154 respondents, for a response rate of 93.5%.

The University of Colorado and University of Indianapolis followed a similar procedure of survey administration as Duke University. The University of Colorado cohorts received an initial letter discussing the non-cognitive survey on May 8, 2017, and the survey opened on May 9, 2017. Students received a reminder email on May 15, 2017, and the survey closed on May 27, 2017. The University of Colorado cohorts had 115 of a possible 136 respondents, for a response rate of 84.6%. Similarly, the University of Indianapolis cohorts received the initial survey on June 8, 2017, with reminders for completion on June 15 and 26, 2017. The University of Indianapolis cohorts had 71 of a possible 83 respondents, for a response rate of 85.5%.

### Data extraction

Qualtrics provided raw identified data in Excel format for each institution. De-identified data were shared at a central location (Duke University) and the data were compiled into a common IBM SPSS ver. 24.0 file (IBM Corp., Armonk, NY, USA).

### Missing values

Initially, 330 surveys were initiated, of which 298 were completed. Complete item data were present in 98.6% of the data. In total, 90.3% of surveys contained complete data. Since there were few missing values, SPSS was instructed to ‘skip’ the missing values.

### Data analyses

SPSS software was used for all analyses (IBM Corp., Armonk, NY, USA). The alpha level was set at P= 0.05 for statistical significance for all analyses. Exploratory factor analysis (EFA) was used to identify subgroups of factors based on responses to the non-cognitive items. For EFA to be appropriate, the following 2 conditions are necessary: (1) a proper sample size and (2) appropriateness of use. Although no consensus exists on the appropriate sample size for factor analysis, our sample of 298 was deemed to be near the threshold of a ‘good’ power estimate [27].

Sample adequacy was assessed via an anti-image correlation matrix, with factors less than 0.5 being excluded from the analysis. Sample adequacy was also assessed through the Kaiser-Meyer-Olkin (KMO) statistic, which is the proportion of variable variance caused by underlying factors. A KMO statistic above 0.50 was deemed acceptable for this analysis [28]. The Bartlett test of sphericity confirmed that the correlation matrix was suitable for structure detection.

Factor selection was determined based on an eigenvalue greater than 1 occurring prior to the inflexion point on a scree plot [29], which has been deemed an appropriate method of factor selection for sample sizes greater than 200 [30]. Once the variables were extracted, and after several iterations were run involving different rotation analyses, factor detection was enhanced using orthogonal (varimax) rotation [31]. Varimax rotation was selected because qualitatively, its results provided the most logical combination of groups. A variable was considered as a defining part of a factor if the factor regression coefficient was greater than or equal to 0.4 [28]. Once common indices were identified, the research team then labeled each item based on the commonality of a latent construct.

## Results

Initiated surveys were captured from 88.5% of the possible respondents, with a total of 330 students, of whom 144 (43.6%) were from Duke University, 115 (34.8%) were from the University of Colorado, and 71 respondents (21.5%) were from the University of Indianapolis. One hundred seventy-three (52.4%) respondents were first-year students and 157 (47.6%) were second-year students. Of the total respondents, 298 (90.3%) completed all elements of the survey. Raw data are available from Supplement 1.

Anti-image correlation resulted in 0 factors being removed, and the KMO statistic of 0.804 confirmed sample adequacy. The Bartlett test (8,196.245, P≤ 0.01) confirmed the suitability of structure detection. During the EFA phase, a total of 29 items were removed because each had a regression coefficient < 0.4, leaving 39 items for dimensional assessment.

The remaining 39 items loaded on 3 axes (Table 2). These 3 axes included the latent constructs of adaptability (16 items), intuitiveness (12 items), and engagement (11 items). The adaptability construct included items from the IUS and the Psychological Flexibility Questionnaire. The intuitiveness construct included items from the Schutte Self Report Emotional Intelligence Test, the MSI scale, and the Grit-S. The engagement construct included items from the IRI and the Psychological Flexibility Questionnaire.

## Discussion

Among first- and second-year DPT students in 3 different programs, we administered items representing multiple non-cognitive constructs (e.g., emotional intelligence, resilience). In doing so, we identified the following three domains: adaptability, intuitiveness, and engagement. The survey questions in the adaptability domain address how well an individual deals with uncertainty. Example statements in the domain include, “When I am uncertain I can’t function very well” and “I am open to experiencing the different and the exceptional.” An individual’s empathy, emotional intelligence, and ability to be open-minded can be explored within the intuitiveness domain. Example statements in this domain include, “I am aware of my emotions as I experience them” and “I am able to recognize the wishes of others.” Finally, the engagement domain encompasses one’s ability to show commitment to interpersonal interactions. Example statements from this domain include, “I believe that there are two sides to every question and try to look at them” and “There are usually many possible ways to do things.” These 3 domains represent desirable traits of learners and health care professionals and correspond well to pre-existing personality constructs associated with academic success, such as openness, agreeableness, extraversion, and emotional intelligence [32]. Some of the items removed during EFA involved subscales analyzing happy emotions, social irritability, and use of manipulation in social settings. These subscales could be considered to be at the extremes of non-cognitive traits, which might have been why they did not fit into the dimensional assessment. The 3 domains identified in this preliminary instrument may be ideal for consideration in the admissions process, as well as in future studies of academic and professional success.

DPT programs are training the professional physical therapist workforce. Non-cognitive assessments have shown great value in workforce development, and it has been argued that non-cognitive skills are more valuable to employers than cognitive abilities in predicting job performance [33]. Professionalism is essential for having successful, positive interactions in clinical settings, and professionalism training in DPT curricula requires the investment of significant cost and effort [34,35]. Admissions committees should target individuals who have the ability to flourish in clinical practice and be successful as members of the physical therapy workforce. Accepted students will hopefully be individuals who work well on teams, develop strong therapeutic alliances with their patients, and seek leadership positions, but also have academic success, meet their goals, and enjoy their careers long-term.

In an attempt to better identify the desired traits of prospective students, many health professions have initiated a holistic review process. Holistic admissions reviews are an individualized process that aims to capture an applicant’s full breadth of potential. Such reviews continue to weigh a prospective student’s cognitive metrics, but have also increasingly focused on applicants’ experiences and attributes to build a more diverse and collaborative workforce. Holistic review processes have been implemented across the health professions of medicine, dentistry, nursing, and physical therapy [36,37,38]. It has been argued that personality traits should be used to predict academic success in higher education [39]. However, the desired traits can be difficult to identify due to a lack of standardized metrics.

The 39 items included in our preliminary survey had strong construct validity, dimensionality, and content validity. Our initial removal phase was designed to improve face validity. However, for there to be value in this tool, our next steps going forward will require assessment of concurrent validity with academic performance, National Physical Therapy Exam (NPTE) pass rates, and institution-specific alumni milestones. This instrument will require continued evolution as DPT programs examine survey results. The researchers at each institution in this study plan to compare respondents’ completed survey findings with their future academic success in both the didactic and clinical portions of the curriculum. The survey results will also be compared with the pass rate on the NPTE and program-specific alumni milestones. The potential use of this tool for admissions decisions has yet to be established. Furthermore, our sample included current DPT students who had already matriculated into a program. Neissen et al. suggested that individuals may respond differently to questionnaires when high-stakes scenarios such as graduate school admissions are involved [40]. There are also inherent difficulties with self-reported measures, as individuals may not answer surveys honestly as they try to determine the desired responses. Furthermore, if such a survey were to be used in admissions decisions, there is the potential that prospective students may seek coaching to become more attractive candidates.

Each institution has unique priorities for the desired traits of prospective students, and the proposed non-cognitive evaluation tool will hopefully enable programs to improve their ability to identify prospective learners who correspond with their desired traits. This survey could play an important role in addressing non-cognitive traits of prospective learners in a holistic admissions review process. Ideally, traits could be identified that would correlate with academic and clinical success to better identify individuals during the admissions process who reflect the unique characteristics of each program.

## Figures and Tables

**Table 1.** Demographic information of the respondents and response rate

Cohorts	Total #	# Respondents	% Response	Male	Female	% Female	Average age (yr)
University of Colorado (second-year cohort)	70	53	76	14	56	80.00	24
University of Colorado (first-year cohort)	66	62	94	16	50	75.80	24
Duke University (second-year cohort)	74	72	97	24	50	67.60	23
Duke University (first-year cohort)	80	72	90	25	55	68.80	22
University of Indianapolis (second-year cohort)	40	32	80	16	24	60.00	22
University of Indianapolis (first -year cohort)	43	39	91	9	34	79.10	22
Total	373	330	88	104	269	72.11	

**Table 2.** Survey items and dimensions

Survey statements	Scale	Adaptability coefficient	Intuitiveness coefficient	Engagement coefficient
Uncertainty makes life intolerable.	Intolerance of Uncertainty Scale	0.541		
It’s unfair not having any guarantees in life.	Intolerance of Uncertainty Scale	0.553		
My mind can’t be relaxed if I don’t know what will happen tomorrow.	Intolerance of Uncertainty Scale	0.702		
It frustrates me not having all the information I need.	Intolerance of Uncertainty Scale	0.665		
One should always look ahead so as to avoid surprises.	Intolerance of Uncertainty Scale	0.520		
A small unforeseen event can spoil everything, even with the best of planning.	Intolerance of Uncertainty Scale	0.593		
When I am uncertain, I can’t go forward.	Intolerance of Uncertainty Scale	0.664		
When I am uncertain I can’t function very well.	Intolerance of Uncertainty Scale	0.681		
I always want to know what the future has in store for me.	Intolerance of Uncertainty Scale	0.625		
I should be able to organize everything in advance.	Intolerance of Uncertainty Scale	0.574		
Being uncertain means that I lack confidence.	Intolerance of Uncertainty Scale	0.602		
I think it’s unfair that other people seem sure about their future.	Intolerance of Uncertainty Scale	0.463		
The ambiguities in life stress me.	Intolerance of Uncertainty Scale	0.687		
I can’t stand being undecided about my future.	Intolerance of Uncertainty Scale	0.666		
I am open to experiencing the different and the exceptional.	Psychological Flexibility Questionnaire	-0.465		
At times I can make significant decisions, based on my need to change.	Psychological Flexibility Questionnaire	-0.446		
I know when to speak about my personal problems to others.	Schutte Self Report Emotional Intelligence Test		0.436	
I am aware of my emotions as I experience them.	Schutte Self Report Emotional Intelligence Test		0.410	
By looking at their facial expressions, I recognize the emotions people are experiencing.	Schutte Self Report Emotional Intelligence Test		0.588	
I am aware of the non-verbal messages other people send.	Schutte Self Report Emotional Intelligence Test		0.607	
I know what other people are feeling just by looking at them.	Schutte Self Report Emotional Intelligence Test		0.546	
I can tell how people are feeling by listening to the tone of their voice.	Schutte Self Report Emotional Intelligence Test		0.519	
It is difficult for me to understand why people feel the way they do.	Schutte Self Report Emotional Intelligence Test		-0.440	
I am able to recognize the wishes of others.	Measuring Social Intelligence		0.482	
I know how to act in accordance with the feelings of others.	Measuring Social Intelligence		0.530	
I am able to guess the feelings of others even when they do not want to show them.	Measuring Social Intelligence		0.539	
In contact with other people I can recognize their intention.	Measuring Social Intelligence		0.450	
I have been obsessed with a certain idea or project for a short time but later lost interest.	Short Grit Scale		0.405	
I really get involved with the feelings of the characters in a novel.	Interpersonal Reactivity Index			0.585
I am usually objective when I watch a movie or play, and I don’t often get completely caught up in it.	Interpersonal Reactivity Index			-0.405
I try to look at everybody’s side of a disagreement before I make a decision.	Interpersonal Reactivity Index			0.419
Becoming extremely involved in a good book or movie is somewhat rare for me.	Interpersonal Reactivity Index			-0.429
After seeing a play or movie, I have felt as though I were one of the characters.	Interpersonal Reactivity Index			0.602
I believe that there are two sides to every question and try to look at them both.	Interpersonal Reactivity Index			0.550
When I watch a good movie, I can very easily put myself in the place of a leading character.	Interpersonal Reactivity Index			0.611
When I’m upset at someone, I usually try to ‘put myself in his shoes’ for a while.	Interpersonal Reactivity Index			0.422
When I am reading an interesting story or novel, I imagine how I would feel if the events in the story were happening to me.	Interpersonal Reactivity Index			0.617
There are usually many possible ways to do things.	Psychological Flexibility Questionnaire			0.445
Reality has many different aspects.	Psychological Flexibility Questionnaire			0.437

## Supplementary materials

Supplement 1. Data files are available from https://doi.org/10.7910/DVN/Y75CP2

Supplement 2. Abstract recording
